# Tackling anti-coagulation under-prescription in the elderly

**DOI:** 10.18632/aging.101809

**Published:** 2019-02-01

**Authors:** Ameenathul M. Fawzy, Tse-Fan Chao, Gregory Y.H. Lip

**Affiliations:** 1 Liverpool Centre for Cardiovascular Science, University of Liverpool and Liverpool Heart & Chest Hospital, Liverpool, United Kingdom; 2 Division of Cardiology, Department of Medicine, Taipei Veterans General Hospital, Taipei, Taiwan; 3 Institute of Clinical Medicine, and Cardiovascular Research Center, National Yang-Ming University, Taipei, Taiwan

**Keywords:** atrial fibrillation (AF), stroke risk factors, warfarin, oral anti-coagulation (OAC), stroke prevention

Atrial fibrillation (AF) is an age-associated common arrhythmia, with a prevalence increasing from <0.1% to 10% in individuals aged ≤55 and ≥80 years respectively [1]. Currently, the over-80s population is growing fastest and projected to increase from 137 to 425 million over the next 30 years [2]; a change that will result in a further rise in AF prevalence. The focus therefore needs to shift towards enforcing measures that will alleviate the disease burden which will undoubtedly follow. In this regard, a key focus in AF management is stroke prevention with oral anti-coagulation (OAC) [3].

For the elderly patient, the risk of stroke is amplified by the combination of age and AF; both which are independent risk factors for stroke. Having AF results in a 5-fold increase in the risk, which at the age of 80-84 years is about 23% [4].

Disappointingly, a significant proportion of the elderly remain non-anticoagulated. Under-appreciation of stroke risks and over-estimated bleeding risks go hand-in-hand at influencing this. Elderly patients are also disadvantaged by a lack of uniformity in physicians’ perceptions and (until recently) guidelines, owing to limited data from randomised controlled trials specific to the elderly. Also, OAC therapy is partly dependent on the experience and views of the physician.

The presence of age-related factors such as frailty, polypharmacy and co-morbidities like dementia confound matters by impugning patients’ abilities to comply with or safely tolerate the perceived ‘high-risk treatment’. Not only this but because these factors are associated with falls, there is much fear about bleeding complications, which prevents physicians from initiating treatment. As a result, many are prescribed agents like aspirin as a middle ground, which have been proven ineffective at preventing AF-related strokes [5].

Time and again, questions are raised about the overall benefit of OAC in the elderly given concerns over bleeding. With the largest observational study of this cohort, Chao et al. [6] addresses this by comparing the risk of ischaemic stroke to the risk of intracranial haemorrhage (ICH) in Taiwanese patients ≥90 years, to determine the net clinical benefit (NCB) of OAC. Two cohorts were studied; one from the pre-NOAC era (1996-2011) and the other from the NOAC era (2012-2015). For the former group, these risks were compared between patients with (n=11064) and without (n=14658) AF, neither on anti-thrombotic therapy. After a mean follow-up of 2.06±2.15 years, a significantly elevated ischaemic stroke risk was identified in the AF group. Stroke rates were not significantly different between AF patients on anti-platelet agents and not on anti-thrombotic therapy, but stroke risk was much lower for the sub-group taking warfarin. ICH risk was similar across all groups, and warfarin was associated with a positive NCB.

In the second cohort, stroke risks were similar between patients on warfarin (n=768) and NOACs (n=978) but ICH risk was substantially lower with the latter. An increase in the uptake of OACs was seen from 3.9% to 16.1% (7.1% on warfarin and 9% on NOACs) between the two eras. Proposed reasons included the relative efficacy, safety and convenience of NOACs and increasing awareness of stroke prevention in AF.

Although the numbers of patients on OAC and events were small, the findings by Chao et al add validity to the growing evidence supporting OAC use in the elderly. Thus, it is time for a more proactive approach to anti-coagulation (Figure 1). In one prospective study, physician-cited reasons for withholding warfarin included older age, cognitive impairment and previous haemorrhage [7]. The authors concluded that many elderly patients were not optimal candidates for warfarin, calling out for alternative stroke prevention strategies; however, NOACs have since overcome many of the issues presented by warfarin. In another systematic review of physicians’ perceptions towards OAC, perceived uncertainty or limited evidence, the need for individualised decision making and feelings of delegated responsibility were reported as primary concerns [8].

**Figure 1:**
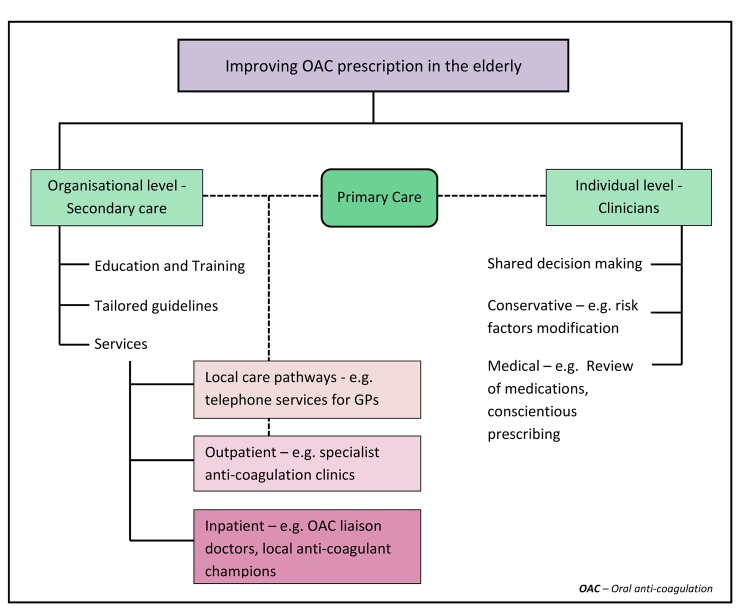
Outlines measures that can be taken to improve oral anti-coagulation prescription rates in elderly patients with atrial fibrillation.

At an organisational level, this emphasises a need for implementation of tools to facilitate communication between primary and secondary care as well as relevant sub-specialties such as general medicine, cardiology and geriatrics. Establishing local guidelines tailored for the elderly and services which have a geriatrician in place as an anti-coagulation liaison; to provide input and perform comprehensive geriatric assessments on complex patients would be of value. More education of healthcare professionals is imperative to ensure treatment is not denied for reasons such as age and frailty.

Taking into consideration bleeding risk assessment, there are plenty that clinicians can do on an individual basis to modify risk-factors for bleeding. Simple measures such as provision of visual and mobility aids, advice on alcohol abstinence and steps to counter postural hypotension could go a long way for those with a predisposition to falling. Conscientious prescribing to minimise polypharmacy and concurrent use of medications such as non-steroidal anti-inflammatory agents, optimising blood pressure and educating patients on the importance of OAC can be undertaken without specialist input. Where possible, clinicians should aim to modify risk factors to facilitate OAC prescription, rather than using them to rationalise OAC disuse.

## References

[1] Chugh SS, et al. Circulation. 2014; 129:837–47. 10.1161/CIRCULATIONAHA.113.005119 24345399 PMC4151302

[2] United Nations. Department of Economic and Social Affairs PD. World Population Prospects. 2017

[3] Lip GY, et al. Thromb Haemost. 2017; 117:1230–39. 10.1160/TH16-11-087630457156

[4] Marinigh R, et al. J Am Coll Cardiol. 2010; 56:827–37. 10.1016/j.jacc.2010.05.028 20813280

[5] Hart RG, et al. Ann Intern Med. 2007; 146:857–67. 10.7326/0003-4819-146-12-200706190-00007 17577005

[6] Chao TF, et al. Circulation. 2018; 138:37–47. 10.1161/CIRCULATIONAHA.117.031658 29490992

[7] Hylek EM, et al. Stroke. 2006; 37:1075–80. 10.1161/01.STR.0000209239.71702.ce 16527999

[8] Mas Dalmau G, et al. BMC Fam Pract. 2017; 18:3. 10.1186/s12875-016-0574-0 28086887 PMC5234257

